# Patient-Defined Cultural Safety in Perinatal Interventions: A Qualitative Scoping Review

**DOI:** 10.1089/heq.2023.0152

**Published:** 2024-03-13

**Authors:** Emilie E. Egger, Bridget Basile Ibrahim, Kate Nyhan, Mukund Desibhatla, Dara Gleeson, Ashley Hagaman

**Affiliations:** ^1^ Department of Social and Behavioral Sciences, Yale School of Public Health, New Haven, Connecticut, USA.; ^2^ Yale University School of Nursing, Orange, Connecticut, USA.; ^3^ Cushing/Whitney Medical Library, Yale University Environmental Health Sciences, Yale School of Public Health, Yale University, New Haven, Connecticut, USA.

**Keywords:** cultural safety, perinatal care, patient satisfaction, qualitative research, scoping review

## Abstract

**Problem:**

Cultural safety is an approach to patient care designed to facilitate respect of patients' cultural needs and address inequities in care in culturally diverse situations.

**Background:**

Much literature considers culturally safe care during the perinatal period, yet little is known about how patients experience and understand cultural safety. This is despite patient-defined care being one of the definitions of cultural safety.

**Question, Hypothesis, or Aim:**

This scoping review investigates what is known from existing qualitative literature about patients' experience of cultural safety frameworks in perinatal interventions.

**Methods:**

A search for “cultural safety” OR “culturally safe” in PubMed, Ovid Medline, Ovid Embase, Cumulated Index to Nursing and Allied Health Literature, Scopus, Scielo, and Latin America and the Caribbean Literature on Health Sciences returned 2233 results after deduplication. Title–abstract and full-text screenings were conducted to identify qualitative studies of cultural safety from perinatal patients' perspectives. Seven studies were included in the final analysis. Data were open coded using NVivo.

**Findings:**

Three themes were identified: (1) care that acknowledged that their lives were different from patients in the dominant culture, (2) receiving care in community, and (3) care providers who respected their choices and culturally specific knowledge.

**Discussion:**

This research shows how cultural safety intersects with other equity-based frameworks used in midwifery and obstetrics.

**Conclusion:**

Building on this research could lead to new protocols that address complex social and physical needs of marginalized people during the perinatal period.

## Objective

To determine what is known from existing qualitative literature about patients' experience of cultural safety frameworks in perinatal interventions.

## Introduction

**Table:** 

**Problem or issue:**	Despite widespread calls for culturally safe care, definitions of a culturally safe intervention are not well researched.
**What is already known:**	According to the definition of cultural safety, patients must define culturally safe care. Growing evidence suggests culturally safe care is correlated with better perinatal and birth outcomes.
**What this article adds:**	This article adds patient understanding of what makes culturally safe care. It makes clear the need for more research on this topic and provides foundation for culturally safe interventions during the perinatal period and birth.

Cultural safety is an approach to health care that grew out of demands of Māori people in the 1980s and 1990s to redress historical and persistent mistreatment they had experienced and continued to experience while receiving care in New Zealand/Aotearoa while receiving care in New Zealand/Aotearoa. This new framework emphasized reflection by health care providers of their roles in perpetuating health inequities within socially inequitable societies.

Rather than a checklist or set of guidelines designed to pertain to encounters between specific populations, cultural safety can best be described as an approach to care adopted by health care providers to ensure that all their patients, no matter what marginalized background they come from, are treated with respect and receive equitable care. The first article on cultural safety defined it as the “effective nursing of a person/family from another culture by a nurse who has undertaken a process of reflection on their own cultural identity and recognises the impact of [their] own culture on nursing practice.”^1^

Cultural safety has several characteristics in common with other culturally aware health frameworks, such as cultural competency and cultural awareness, yet it differs from these frameworks in several key ways.^2^ The most commonly used definition of cultural competency is “a set of congruent behaviours, attitudes, and policies that come together in a system, agency, or among professionals and enable that system, agency, or those professionals to work effectively in cross-cultural situations.”^3^ This framework emphasizes skills, knowledge of other cultures, and focuses on the individual level of care.

Along with cultural awareness, cultural competency has been criticized as being too limited, due to its emphasis on individual competencies rather than structural inequalities.^4^ In contrast, cultural safety acknowledges provider power imbalances and emphasizes the ability of care providers to continuously reflect on the power they hold in patient encounters and how they can remedy these imbalances across both structural and individual levels within inequitable societies.^2^ Another key distinction is that cultural safety requires that patients themselves determine whether or not the care they receive is culturally safe.^1^

This speaks of the way cultural safety de-emphasizes specific behaviors and instead relies on continual responsiveness to patients' needs and also differentiates cultural safety from cultural humility, with which it shares several attributes.^5^ Foronda and colleagues define cultural humility as “a process of openness, self-awareness, being egoless, and incorporating self-reflection and critique after willingly interacting with diverse individuals.”^5^

Health systems in additional countries such as Australia and the United Kingdom have adopted cultural safety as a framework for health interventions, especially in nursing and midwifery contexts.^6^ For example, in 1992, the Nursing Council of New Zealand instituted a cultural safety training requirement for all nurses and midwives in the country, although different programs cite varying protocol and rates of training.^7^

Cultural safety was developed to redress the effects of settler colonialism on Indigenous people and other marginalized people. The theoretical justification for incorporating this framework into care protocols in all regions is addressing the fundamental power differences in a clinic or health system will uproot other health disparities. With this focus on responsiveness to patient needs, cultural safety also consolidates the tenets of other prominent frameworks used in midwifery and obstetric care, such as patient-centered care, the Quality Maternal and Newborn Care Framework, and High-Quality Health Systems.^8–10^

Beyond societies with a settler-colonial history, this framework can be applied because it orients health staff to confront all kinds of power dynamics that the health care environment is replicating within a given society or community, leading toward what West and colleagues have termed “ethical relationality.”^11^

Cultural safety is a framework on the path toward cultural security. Cultural security focuses on enabling effective communication between patients and providers from different cultural backgrounds and to create an environment in which patients' cultural beliefs and rights are not compromised.^12^ At this time, very few studies on patients' perceptions of cultural security exist. Creating a more robust patient-generated definition of cultural safety could lead to more insight as to how to develop culturally secure care. One goal of this review is to lead to a better understanding of culturally safe interventions to enable more culturally secure interventions in the future.

We chose to investigate culturally safe interventions during the perinatal period because of the continued high risk for morbidity and mortality to women during pregnancy, birth, and postpartum. Moreover, due to the central criterion within cultural safety that patients decide whether the care they receive is culturally safe or unsafe,^1^ we focused on interventions that explicitly implemented culturally safe care and studies in which people who were pregnant or had recently given birth were explicitly asked questions about what made the care they received culturally safe or not. Although many articles included in title–abstract review included themes of cultural safety in their analyses, we kept only those articles in which women were asked to define cultural safety qualitatively to understand how such care is experienced.

The focus of qualitative research on asking open-ended questions to ask how and why processes occur makes it especially suited for our research question.^13^ Although many of these articles asked the question “Is the intervention culturally safe?” our review focused on defining “What is a culturally safe intervention?” This scoping review complements existing literature on cultural safety as we seek to address how women in the perinatal period conceptualize cultural safety to aid the development of culturally safe perinatal interventions. This is the first literature review to analyze this aspect of the framework.

The review will be useful to establish evidence about effective cultural safety initiatives, which a recent review noted were “undermined” by a “lack of evidence.”^14^ Recent research has included validating tools and evaluations of culturally safe interventions,^15^ as well as providers' knowledge of the framework.^16^ This review's focus on birthing people's experience of perinatal interventions complements studies of nurse and midwife cultural safety training and perceptions of culturally safe care in their fields.^17–21^ Although some syntheses focusing on cultural safety have focused on the needs of certain marginalized populations, none of these addressed perinatal care specifically from the patient perspective.^22–25^

To understand how pregnant, birthing, and postpartum people experience cultural safety during health care encounters during the perinatal period, this scoping review aims to identify and synthesize qualitative studies that elaborate on the following research question:

### What is known from existing qualitative literature about how patients understand culturally safe perinatal interventions?

This is a question that qualitative research, with its focus on how and why, is best suited to answer.^26^ We set out to conduct a meta-synthesis^27^ of qualitative data to further understand cultural safety and how it relates to other frameworks focused on delivering maternity care. Although no numerical benchmark exists for studies necessary for a meta-synthesis, because so few studies remained after screening, we did not have sufficient information to theorize around cultural safety in perinatal health interventions “across groups” and did not have familiarity with the original authors and their work to put forward a comprehensive theory on cultural safety.^28^

Our search criteria, although we believe to be rigorous and as complete as possible, are not comprehensive enough to cover all knowledge that perhaps implicitly addresses cultural safety in perinatal interventions or in which patients define culturally safe interventions, but where those are not the central purpose of the study (Thorne). Our review is, therefore, a scoping review, which “identifies common themes”^28^ and identifies gaps in the literature on this topic and that calls for a more robust literature and inclusion of other kinds of knowledge on cultural safety.

This article uses the terms women and pregnant people in an endeavor to be as inclusive as possible using prenatal, birthing, and postpartum health services. When citing a specific study, we have retained the term used in that study.

## Inclusion Strategy

A scoping review was conducted using the Population/Problem/Patient; Intervention/Issue; Outcome framework.^29^ The population was individuals who were pregnant to 1-year postpartum, the intervention under investigation was cultural safety-guided interventions, and the outcome was how patients experience cultural safety as measured by qualitative studies. A scoping review was appropriate to this question because of the heterogeneity of studies that did not present recommendations in a uniform way. No protocol was published.

## Methods

### Search strategy

In consultation with a medical librarian (K.N.), a literature search was completed in the following databases: PubMed, Ovid Medline, Cumulative Index to Nursing and Allied Health Literature Complete (CINAHL), Latin American and Caribbean Health Sciences Literature (LILACS), Scielo, Ovid EMBASE, and Scopus using the terms [cultural safety OR culturally safe] in fields including (depending on database features) titles, abstracts, author keywords, and subject indexing (Table 1).

**Table 1.** Search Strategy

PubMed	Cultural safety [tw] OR culturally safe^*^ [tw]
Ovid Medline	(Cultural safety OR culturally safe^*^).mp.
Ovid Embase	(Cultural safety OR culturally safe^*^).mp. or cultural safety/
CINAHL	TI (“Cultural safety” OR “culturally safe^*^”) OR AB (“Cultural safety” OR “culturally safe^*^”) OR MH (cultural safety)
Scopus	TITLE-ABS-KEY (“Cultural safety” OR “culturally safe^*^”)
Scielo	“cultural safety” OR “seguridad cultural”
LILACS	“cultural safety” OR “culturally safe”

The research team tested a more complex search that included additional terms for the population of interest and qualitative methods. However, the simple search strategy described above was ultimately used because it was more sensitive to the complexity of our research question, which asked how participants defined cultural safety. Some articles engaged with cultural safety in great detail,^30^ but did not include patient perspectives; others did not mention whether patients were asked specifically about cultural safety until the results section. Therefore, full-text screening was necessary to discern whether the studies fulfilled inclusion criteria.

### Study selection

Qualitative studies published in English or Spanish, the languages the primary author reads, after 1988 (the year the cultural safety framework was developed) were included. Studies were single screened in Covidence. Studies were screened at the title–abstract level to determine whether the women participating in the studies defined cultural safety in perinatal interventions and whether they were conducted using qualitative methods. Although many articles included researchers determining whether cultural safety was achieved,^25^ they were excluded because this review focused on pregnant and postpartum people defining cultural safety.

Studies that considered perinatal care from the perspective of marginalized groups were not included unless participants were explicitly asked about cultural safety or about an intervention designated as culturally safe. Other studies of perinatal care made suggestions for applying findings to achieve culturally safe perinatal care. However, these studies were excluded as they did not directly pertain to our research question.

### Data extraction and analysis

The JBI Qari Data Extraction Tool was used to collect information from each included study (Table 2).^31^ The results sections of seven articles were included for coding, which was conducted using NVivo software.^32^ The primary author developed a codebook using inductive coding, based on *in vivo* codes of participants' discussion of their experiences within health care systems through iterative analysis that always centered the research question.^26^ The primary author then applied codes to the seven articles.

**Table 2.** Data Extraction Instrument

**Title**	Conceptualizing cultural safety at an Indigenous-focused midwifery practice in Toronto, Canada: Qualitative interviews with Indigenous and non-Indigenous clients	Conception of a Resource: Development of a physical activity and healthy living resource with and for pregnant urban First Nations and Metis women in Ottawa, Canada	Australian Aboriginal Kinship: A means to enhance maternal well-being	Having a Quiet Word’: Yarming with Aboriginal Women in the Pilbara Region of Western Australia about Mental Health and Mental Health Screening during the prenatal period	A postcolonial feminist discourse analysis of urban Aboriginal women's description of pregnancy-related weight gain and physical activity	Aboriginal and Torres Strait Islander women's experiences accessing standard hospital care for birth in South Australia—A phenomenological study	An Affront to her Mana: Young Māori Mothers' Experiences of Intimate Partner Violence
**Date**	September 29, 2020	2017	June 1, 2011	November 1, 2019	2016	January 14, 2016	July 2021
**DOI**	10.1136/bmjopen-2020-038168	10.1080/2159676X.2016.1246471	10.1016/j.wombi.2010.06.003	10.3390/ijerph16214253	/10.1016/j.wombi.2015.08.003	10.1016/j.wombi.2016.01.004	10.1177/0886260518815712
**Authors**	Churchill ME, Smylie JK, Wolfe SH, Bourgeois C, Moeller H, Firestone M^30^	Darroch FE, Giles AR^39^	Dietsch E, Martin T, Shackleton P, Davies C, McLeod M, Alston M^36^	Carlin E, Atkinson D, Marley JV^37^	Darroch FE, Giles AR^40^	Brown AE, Fereday JA, Middleton PF, Pincombe JI^38^	Dhunna S, Lawton B, Cram F^41^
**Journal**	*BMJ Open*	*Qualitative Research in Sport, Exercise and Health*	*Women and Birth*	*International Journal of Environmental Research and Public Health*	*Women and Birth*	*Women and Birth*	*Journal of Interpersonal Violence*
**Methods**	Semistructured interviews (*n*=20)	Focus groups of 20 (*n*=3); semistructured interviews (*n*=5)	Semistructured interviews (*n*=3)	Semistructured interviews (*n*=15)	Semistructured interviews and focus groups (*n*=25)	Semistructured interviews (*n*=14)	Semistructured interviews (*n*=43)
**Population and location**	Urban, Indigenous-focused midwifery clinic in Toronto, Canada	Pregnant, postpartum urban First Nations and Metis women in Ottawa, Canada	Postpartum women who had to leave their rural communities to give birth in New South Wales, Australia	Women who had experienced the intervention during the perinatal period in Pilbara Region, Australia	Pregnant and postpartum Aboriginal women in Ottawa, Canada	Postpartum Indigenous women who had given birth at a large tertiary teaching hospital in South Australia	Māori women aged 14–19 years in Wellington, Hawkes Bay, New Zealand/Aotearoa
**Intervention**	Antenatal care and delivery services	Healthy living resource	Mental health services	Mental health screening and services	Healthy living resource	Delivery services	Domestic violence interventions
**Definition of cultural safety used by authors**	“culture is …fluid…integral to social structuring knowledge systems, and relationships”; “CS is both process and outcome”; “CS dictates that the only person who can truly define whether a service is culturally safe is the person receiving that service.”		Provider “is aware and sensitive to the needs of women, they legitimise and value cultural differences and enable women themselves to define the nature of a culturally safe relation-ship.”	No definition included	No definition included	“…something which can only be determined by the recipient of the care, and which requires health care providers to examine their own cultural identities, attitudes, beliefs and the power balance within the health care relationship”	“…a concept popularized and standardized by Māori nurses in 1988 to respond to health disparities between Māori and non-Māori by understanding the influence of historical, political, and socioeconomic contexts.”
**How participants defined cultural safety**	Respect and support for choices; personalized continuous relationship with midwives, being different from negative experiences, feeling informed about pregnancy, birth, and the postpartum period, having access to Indigenous knowledge and protocols, feeling at home in practice, relationships interconnected with physical spaces,		Support of kin to combat loneliness, alienation; mutual anger with midwives; long-term emotional morbidity; racism; …	“A lived experience”; connectedness and support; yarning safely	Aboriginal women have different pregnancies than non-Aboriginal women; shame and blame; need for culturally safe resources;	“Knowing what is best and wanting the best for my baby”; “Communicating my way”; “How they made me feel”; “All of my physical needs were met”; “We have resilience and strength despite our hardships”; “Recognising my culture”	Lived realities of young Māori mothers; service responsiveness to intimate partner violence
**Examples**		“So many times we get stuff that is aimed at the Aboriginal community and no one asked us.” We are like, “What the hell is this?” (163)—“There is a strong desire for women to return to their culture during pregnancy” (163)—“Aboriginal people want this [smartphone]—this is how they stay in touch with each other” (163).	“…it was like a depressing thing just come over me instantly. it is so, it's like you can feel it's haunted, man…We all agreed. It's just that feeling.”	“Clinics need to talk these things through…they really do, but they need to build up the friendship and trust…We are coping with so much loss. So much sadness….You have to be really clear with the mum that this is for mum to help her stay strong and look after bub. It is not for DCP [Department of Child Protection[or anyone else, only the midwife and maybe the doctor.”	“…if they want to fix the problems, they need to at least acknowledge our background in order to provide culturally sound care.”; “If I was to see an [image of an] Indigenous woman being strong and active during her pregnancy…I would have been so inspired to see that. But I didn't.”	“How they made me feel”: “Just feel welcome, and not judged…” “Recognising my culture”: “…the first person that has ever, um, asked about respecting the wishes of my Aboriginal, and I was, I was shocked about it and I was, I was amazed and that was a good feeling…”	“She believes me and everything, and she knows I'm good cause I always look after the baby.”; “There is no one for me to, yeah, cause they won't tell me any information about any counseling or any relationship things….”
**Funding sources**	“This study was nested within a larger evaluation study that was supported by a grant from the Ontario Ministry of Health and Long-Term Care.”	N/A	“This study was funded by the Nurses and Midwives Board of New South Wales.”	“This research is funded through a National Health and Medical Research Council Partnership Grant (APP1132659).”	N/A	N/A	N/A

Theme development was based on participants' experiences of health care interventions designated as culturally safe and were grouped into themes with consultation with other authors and cultural safety literature. We noted especially when codes were repeated many times.^33^ Codes were grouped based on similar content and linked to segments of text from the included articles.^33^ The primary author wrote descriptions of themes to analyze how each code fits into each and supported these themes with quotations from the analyzed articles.^34^ The primary author then used this information to write themes that formed the results section.

### Reflexivity statement

Our team consisted of academics in the United States (trained in history of medicine, public health, anthropology, and nursing), each of whom related to the material differently. Several of us have experience giving birth while having cultural needs and advocating for ourselves or vulnerable loved ones in health contexts. None of us are Māori people, and, therefore, had cultural distance from the experiences of the Māori nurse-scholars who developed the term cultural safety. We addressed this distance by staying close to the patient-reported data and thereby close to the Māori-developed definition of cultural safety, which is to hear from patients themselves whether cultural safety was achieved during an encounter and what culturally safe elements of that care included.

Indeed, this definition formed our research question: how do patients in perinatal health encounters define culturally safe care? The participants in the seven included studies were all marginalized by their race and ethnicity in their majority cultures, and the majority were conducted with participants who were themselves Māori or Indigenous people from what is now New Zealand and Australia, and Canada.

A main reason that the number of included studies is so low is that so few studies center the thoughts of patients on this issue and, therefore, do not abide by the Māori definition of cultural safety. Although we believe this to be the first review of its kind based on our extensive search, we consider this article a collaborative contribution toward more understanding about culturally safe care, rather than as proposing a systematic objective definition.

## Results

The initial database search returned 2233 results after deduplication. Title and abstract screening yielded 68 studies and full-text screening yielded 7 studies (Fig. 2). Results are presented according to the Preferred Reporting Items for Systematic Reviews and Meta-analyses (PRISMA) extension for scoping review flow diagram.^35^ Each of the included studies was based on interviews or focus groups with Aboriginal populations in Australia, Canada, or New Zealand. Some focused on the participants' birthing experiences;^30,36–38^ others discussed prenatal interventions.^39,40^

One article sought views of participants from a domestic violence intervention program.^41^ Participants experienced cultural safety across three main themes:^1^ care that acknowledged difference,^2^ being cared for in community, and^3^ respect for choices and community-based knowledge. The following section describes their experiences of culturally safe or unsafe care.

### Care that acknowledged difference

Participants expressed the need for health providers who acknowledged that women's experiences while receiving care were different than women's experiences from the dominant culture and that their care should reflect that difference. This was preferable to not asking about one's cultural needs while giving birth, concealing difference by not speaking about it.

Several women expressed that receiving care that addressed their distinct experiences as a minority within the dominant society, or in which they were asked about their culturally specific needs, was not the norm during their pregnancies and births. An Aboriginal Australian woman said that she was surprised when a provider asked her about her cultural needs and that she experienced this positively:

…the first person that has ever, um, asked about respecting the wishes of my Aboriginal, and I was, I was shocked about it and I was, I was amazed and that was a good feeling…^38^

Although some participants said they did not want to be treated with different protocols than birthing people from the dominant culture, they maintained the importance of being asked whether they needed accommodations. Participants repeatedly spoke of “acknowledgment” of difference as a basic requirement for culturally safe care. One Australian Aboriginal woman explained that not being asked about her culture made her well-being feel like less of a priority for her providers:

I find they [healthcare professionals] do a whole lot of talking about us. How much we gain weight and gestational diabetes and blah, blah, blah. But they never focus any of their care around us and our culture or our communities. …if they want to fix the problems, they need to at least acknowledge our background in order to provide culturally sound care.^40^

Building on this theme, participants noted that care tailored to members of their community must be developed in consultation with members of that community. This communication between health staff from the dominant culture and pregnant and birthing women who are marginalized was key to providing culturally safe care after the first step of asking whether a patient needed a specific accommodation.

So many times we get stuff that is aimed at the Aboriginal community and no one asked us. We are like, “What the hell is this?”^39^

Culturally safe interventions developed alongside the community were more likely to be adhered to by pregnant and birthing women and to become new standards of care. Input from the community was crucial to creating protocols for maintaining health and well-being during the perinatal period that marginalized members of the community came to rely on. One woman explained, “Aboriginal people want this [smartphone]—this is how they stay in touch with each other.”^39^

Recognition of difference also led to individualized care in which women felt valued, respected, and that their needs were being met because their providers understood and accepted those needs. An 18-year-old Métis woman noted how asking questions about her background and needs led to a better working relationship with care providers, which, in turn, increased trust with her health care team.

Once I was in the room with the midwives… all the attention was on me. Just taking the time to ask any questions or, you know, not make me feel like I was being asked to get in and out as quickly as possible. … I felt like I would be able to build a good relationship with the midwives there.^30^

Recognizing difference also meant acknowledging that many marginalized women were coming from situations of systemic isolation from their loved ones and from familiar surroundings to receive pregnancy care or give birth. Receiving this care often required physically relocating for periods during the pregnancy and took an emotional toll on women who could no longer be near loved ones. One Australian Aboriginal woman who had to relocate to a health center 400 km from her home to give birth described her fear:

Because as soon as I walked in like I had no-one with me, really, and I was really out of my comfort zone. And I was crying, because I was just, you know? I just really wanted my Mum or someone there.^36^

Sometimes the isolation that women felt stemmed not from physical separation from loved ones and community members, but from having to give birth and receive pregnancy care in environments that inspired fear due to forced interactions with health providers and state resources from the dominant culture. A Māori mother described the fear she felt when accessing state resources related to finances and job attainment. She explained that these interactions invoked a deep fear that the state would take away her children.

I don't feel confident at all with [CYFS] cause I just think that they're going to take my kids away from me so I don't like that. With WINZ [state financial benefits], I'm scared to go. I don't like to go by myself cause I don't know sometimes what they say to me and I'm saying, “Pardon, oh no.”^41^

Acknowledging difference was described as the first step toward feelings of mutual respect that prompted women to seek and return for care. Acknowledging difference was also crucial to developing effective health interventions in the community.

### “Companionship” with members of their own community

The cultural differences and differences in how marginalized groups experience and are treated during health care require additional support systems for members of those communities. Participants described the importance of receiving perinatal care alongside people from their community, whether that be their loved ones or health providers from their communities. Although they expressed a variety of reasons for this, several women noted that this kind of community presence was specifically important during the perinatal period due to the many strong traditions and family practices focused on the periods of pregnancy, birth, and postpartum.

One woman expressed that feeling a connection with one's community was especially crucial during pregnancy and birth: “There is a strong desire for women to return to their culture during pregnancy.”^39^

Participants explained that ideally, this important cultural companionship should come from women's personal support system and from their health providers, who should be represented among health staff. A First Nations participant explained that hearing that she shared her midwife's cultural background as a First Nations person made her feel more comfortable giving birth.

It's nice when [the midwives] would share where they're from. [My midwife] said what reserve she's from … and she shared her stories. It made me feel more comfortable in talking to her and sharing my story and going through the journey of giving birth, cuz it's a very personal, highly personal, thing.^30^

The desire for community highlighted that pregnancy and birth are significant life events and that women were likely to build trust with providers who had similar life experiences. After commenting on her shared background with her midwife, the above participant added it was ideal to have members of their communities as their providers:

The ideal is the Aboriginal midwife, just being Aboriginal herself. She understands what it means to be an Aboriginal woman because she's lived that life. … She would know and understand and we'd have that connection. We'd understand each other.^30^

Seeing one's community represented in the health system was especially important due to the many racist and discriminatory actions women reported experiencing at the hands of providers who did not share their cultural background. Although some were disrespectful, many of these encounters became explicitly racist interactions. One participant described how her providers' experience attending births led to a power dynamic in which they felt a lack of respect. “They think they've seen it all and so they know everything, but, yeah. But just the lack of respect and stuff they have towards you.”^36^

Another participant, an Aboriginal Australian woman, described how her community was subjected to additional interventions due to racist perceptions of her ethnic group. She described a family member's experience of being criminally targeted because of her Aboriginal background, an experience several women described across the reviews.

I come from [one of those] communities and that was one of my concerns. My niece had a baby and they tested that baby for drugs strictly because she was from that community. She's never done a drug in her life.^40^

Participants said that experiencing the processes of pregnancy and childbirth within their communities cultivated resilience and strength within themselves. This was an important counterforce to the disrespectful and discriminatory treatment they experienced and inspired confidence in their lives and choices.

Talking about the protective stuff, right, the stuff that keep us going, keeps us strong, that's something. We are living this life the best way we can and for us to hear that. For clinic, my midwife to hear that. Now that is a powerful thing.^25^

For marginalized women, experiencing pregnancy and birthing in community increased comfort during the often isolating experiences of pregnancy and birth and in some cases lessened or prevented actions from health staff that further marginalized birthing people.

### Respect for culture-based choices and knowledge

Beyond acknowledging their different needs for support during pregnancy and birth, participants said repeatedly that it was important to them that providers express confidence that they were successfully navigating these periods for themselves and their babies. At the most basic level, this meant providers accepting their choices about their care. One participant described the importance of feeling like the provider trusted her as connected to her feeling like a “good” patient:

She believes me and everything, and she knows I'm good cause I always look after the baby.^41^

For patients who often experienced discrimination or criminalization because of their ethnic, racial, or class status, a lack of judgment or show of support from a health provider was not only an encouraging gesture, but a sign of safety. This was especially true when birthing people's choices conflicted with standard medical advice. One participant described how she felt safe when her provider presented options for care without judgment and left decisions regarding the care of her baby up to her:

[My midwife was] warm, easy to talk to, she didn't judge me for anything I said. She just… she understood, you know. She didn't, like even though, yes, she had to remind me ‘it's better to breastfeed,’ she never pushed the idea on me, you know what I mean?^30^

Respecting birthing people's choices exceeded toleration and included demonstrating genuine admiration for how they navigated the perinatal period as members of a marginalized group. This admiration was most effective when it was expressed concretely within the health facility. One woman described how she desired to see members of her community held up as exemplars of a healthy pregnancy to counteract cultural stereotypes of unhealthy pregnancy perpetuated by the dominant culture on people of her ethnicity. This was important to her both because it would have made her feel part of the larger birthing community and because it would reflect providers' authentic respect for her culture:

If I was to see an [image of an] Indigenous woman being strong and active during her pregnancy…I would have been so inspired to see that. But I didn't.^40^

Similarly, participants said they wanted providers to respect the knowledge of pregnancy and birth that came from their communities. This meant allowing women to follow prenatal and birthing practices they brought from their own cultural backgrounds and respecting them as beneficial to the health and well-being of themselves and their babies. A First Nations participant explained:

Something that would make me feel more culturally rooted would be the chance to either accept or offer food or drinks. Not just water, but like if there was, I don't know, like a tea station or something. Something that makes me feel like I'm going to my granny's house, you know? Or to my auntie's house, or you know? Like where you're just a cup of tea. (I1, First Nations)^30^

Patients described their differences from the dominant culture as a major factor in whether they received culturally safe care during the perinatal period and birth. Acknowledging these differences showed patients that health staff were trying to accommodate their cultural needs. Experiencing pregnancy and birth with people from their communities also gave birthing people more confidence their needs would be respected, even when friction arose between standard protocols and cultural traditions. Demonstrating concrete respect for such traditions through incorporating them into standard care made pregnant and birthing people feel more comfortable during their medical encounters.

## Discussion

The recent focus on quality-of-care frameworks in efforts to decrease maternal and infant mortality calls for more studies like those included in this review.^42^ This review contributes a new synthesis to guide the design of implementable culturally safe health perinatal interventions, keeping in mind Ramsden's original premise that cultural safety is essential to better health outcomes. Cultural safety incorporates the tenets of several frameworks employed widely throughout the fields of midwifery and nursing, which have yet to be brought together in a unifying schema that highlights the relationship between facilitators of responsiveness to patient needs and alleviating health inequities on all levels of health systems with focus on societal power dynamics that influence these health encounters.

Similar to the Person-Centered Maternity Care scale,^43^ cultural safety is a response to the needs of the patient to be treated with dignity and to exercise autonomy in their care through supportive care, communication, and access to social support. It also provides a mechanism for attaining trust and a sustainable way to avoid stigma and discrimination with its focus on ongoing training in reflexivity on the part of the health provider through the expanding development of tools and measures, such as the Holistic Reflection Assessment Tool and the Ganngaleh nga Yagaleh tool.^11,44^

Like the High-Quality Healthcare Systems Framework,^10^ cultural safety acknowledges the need for better confidence in the health system, but adds complexity to the domain “positive user experience” that, despite being a major part of the framework, is vaguely defined. Because of its focus on how power differentials within a larger culture directly influence health inequities within a health system, cultural safety includes each aspect of the framework's foundations, such as equitable and efficient care, resilient patients and population-specific health needs and clinic-level factors, (“governance”; “platforms”), and workplace issues.

The growing cultural safety literature also provides ways of developing and assessing the quality of care called for in high-quality health systems framework in a way that centers patients. Qualitative research will be especially useful to highlight how patients experience these interventions.

**FIG. 1.**
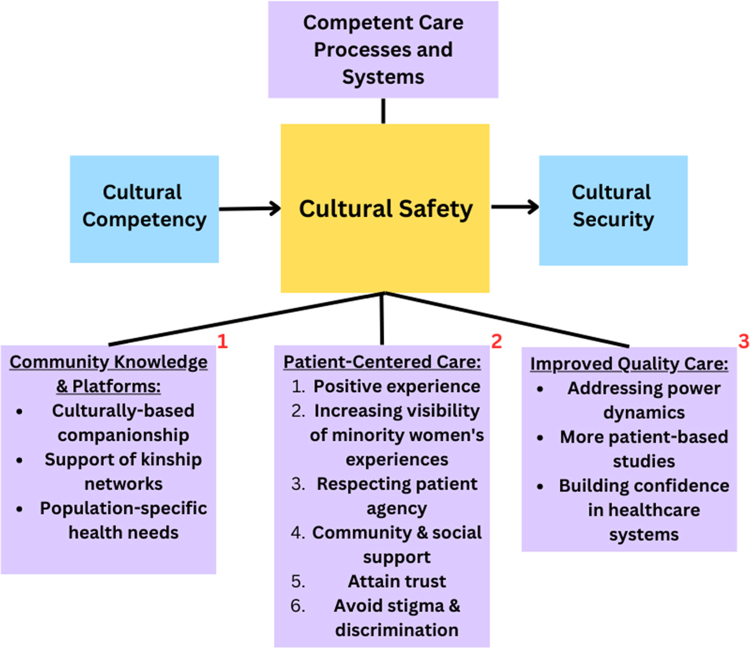
Competent care processes and systems. Cultural safety is defined by (1) Quality Maternal and Newborn Care Framework, (2) Person-Centered Maternity Care, and (3) High Quality Health Care Systems.

As in the Quality Maternal and Newborn Care Framework,^9^ cultural safety framework addresses organizational culture by providing a way to redress problematic power dynamics in the workplace in an ongoing and sustainable way.^45^ It also includes ways to sustain positive relationships and incorporates need for women's knowledge to be recognized while also receiving direction from health staff, as well as the support of their kinship networks (Fig. 1).

**FIG. 2.**
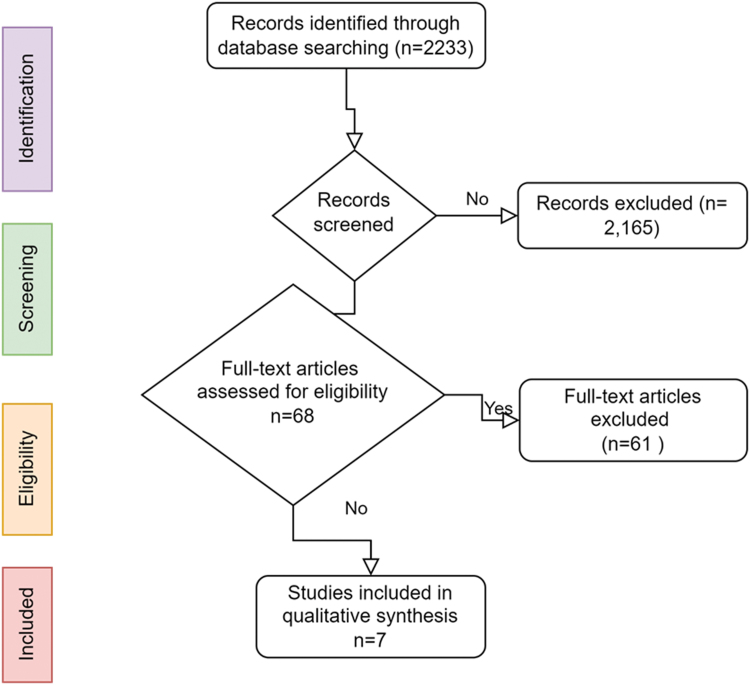
Preferred Reporting Items for Systematic Reviews and Meta-analyses flow diagram.

To highlight the patient agency element of the cultural safety framework, it is critical that future studies address birthing people's experience of cultural safety during their perinatal care. Further research into how women from various marginalized groups experience health care and what they need to feel safe during those experiences is imperative. The ability to provide culturally safe care is a prerequisite for developing culturally secure interventions in which neither patients' rights and needs nor quality health care is abandoned.^12^ Patient perspectives are an essential element of cultural safety training programs.^42^ Additional qualitative research from the patient's perspective is necessary to complement quantitative studies on cultural safety.^24^

The development of standardized cultural safety training is imperative, but further research is required to address differences between students and providers' barriers to providing culturally safe care^11^ and how to standardize cultural safety training across disciplines beyond nursing and midwifery.^11^ Future studies may contribute evidence regarding the link between cultural safety and health outcomes^24^ and the ability for health systems to incorporate systems in which “patients' cultural beliefs and rights are not compromised.”^12^

Validated clinical tools for cultural safety skills include active listening^6^ and reflexivity.^44^ Finally, additional studies on cultural safety will aid in the development of validated evaluation tools, such as the Cultural Safety Survey Scale^15^ and others.^46^

Finally, future scoping and systematic reviews could analyze culturally safe care among specific groups of marginalized patients. Some studies screened for this review addressed the needs of Indigenous women in Australia,^47^ women in Timor-Leste,^48^ and immigrant women in several contexts.^49–51^ Researchers could follow-up on these studies by asking perinatal people about their impression of cultural safety within the health care they received. The work of Indigenous and First Nations scholars should be especially prioritized.

## Conclusion

This scoping review synthesized studies in which perinatal people were asked whether they experienced culturally safe care and to describe that care. Cultural safety is a framework developed to redress the effects of settler colonialism on Indigenous and other marginalized populations. Owing to the potential morbidity and mortality of pregnant people in the perinatal period, it is essential to improve cultural safety interventions for patients during this time to remediate inequities in care. This article reviewed qualitative studies in which pregnant and postpartum people were asked whether they experienced culturally safe care and how they described the care that they received.

Three main themes were identified based on participants' experiences: (1) care that acknowledged differences that encouraged respectful, trustworthy, and effective health care; (2) being cared for with a community connection; and (3) respect for choices and community-based knowledge that accommodated cultural needs and traditions. Addressing cultural safety among various marginalized patient populations necessitates patient-generated definitions to inform the development of culturally safe interventions. Cultural safety involves concepts from other frameworks used in midwifery and nursing that focus on supportive care, confidence in the health care system, and recognizing women's knowledge in maternal care.

The increased visibility of studies like those included in this review may strengthen evidence that culturally safe care is correlated with better perinatal and birth outcomes. Future studies can contribute to fostering the link between cultural safety and health outcomes as well as evaluating culturally safe care given to marginalized people. Further qualitative research can center on the experiences and perceptions of birthing people to complement current studies on nurses and midwives.

## Abbreviation Used

PRISMA: Preferred Reporting Items for Systematic Reviews and Meta-analyses
